# Cost‐effectiveness analysis of the multimodal intervention for dementia prevention: post‐hoc sub‐group analyses of the J‐MINT

**DOI:** 10.1002/alz.086754

**Published:** 2025-01-09

**Authors:** Naoki Takashi, Shosuke Ohtera, Yujiro Kuroda, Hidenori Arai, Takashi Sakurai, J‐MINT study group

**Affiliations:** ^1^ National Center for Geriatrics and Gerontology, Obu, Aichi Japan

## Abstract

**Background:**

This post‐hoc subgroup analysis aimed to estimate the potential cost‐effectiveness of a Japanese multimodal intervention trial for the prevention of dementia (J‐MINT) from a societal perspective.

**Method:**

Using a Markov model, we estimated the economic impact of J‐MINT on disease prevention, drawing on data from the 2019 J‐MINT trial and relevant published literature. The trial, a randomized controlled trial (RCT), focused on participants aged 65 to 85 years with mild cognitive impairment. Participants were randomly assigned either a multidomain intervention group (comprising management of vascular risk factors, physical exercise, nutritional counseling, and cognitive training) or a standard care group (involving vascular risk factor management and receipt of health‐related information every two months). The Markov model consists of five states: at‐risk, mild, moderate, severe dementia, and death. By simulating care costs and quality‐adjusted life years (QALYs), we estimated the incremental cost‐effectiveness ratio (ICER). The simulation spanned from ages 65 to 100, utilizing the Simpson 1/3 rule for correction, with a discount rate of 0.02. The intervention effect was assumed to persist for the remaining lifetime post‐program. Care costs encompassed medical care, public long‐term care, and informal care. The intervention cost was derived from the J‐MINT trial data. In addition to the base case analysis, sensitivity and scenario analysis were conducted to evaluate uncertainty and heterogeneity.

**Result:**

Costs were JPY 18,493,579 and JPY 18,056,997 and QALYs were 12.37 and 12.46 for standard of care and J‐MINT, respectively. This translated to savings of JPY 436,582 (US$ 2,952.01) and QALY gain of 0.09 per person, affirming the dominance of the J‐MINT.

**Conclusion:**

Our estimation supports the potential cost‐effectiveness of multimodal intervention trials, exemplified by J‐MINT, for dementia prevention.

